# Assessment of vascular endothelial growth factor in formalin fixed, paraffin embedded colon cancer specimens by means of a well-based reverse phase protein array

**DOI:** 10.1186/1477-5956-12-27

**Published:** 2014-05-13

**Authors:** Joon-Yong Chung, Till Braunschweig, Seung-Mo Hong, David S Kwon, Soo-Heang Eo, HyungJun Cho, Stephen M Hewitt

**Affiliations:** 1 Tissue Array Research Program & Applied Molecular Pathology Lab., Laboratory of Pathology, National Cancer Institute, National Institutes of Health, Bethesda, MD 20892, USA; 2 Institute of Pathology, RWTH Aachen University, Aachen 52074, Germany; 3 Department of Pathology, Asan Medical Center, University of Ulsan College of Medicine, Seoul 138-736, South Korea; 4 Department of Statistics, Korea University, Seoul 136-701, South Korea

**Keywords:** Vascular endothelial growth factor, Formalin-fixed paraffin-embedded, Colon cancer, Immunohistochemistry, Reverse-phase protein array

## Abstract

**Background:**

Vascular endothelial growth factor (VEGF) is a critical pro-angiogenic factor, found in a number of cancers, and a target of therapy. It is typically assessed by immunohistochemistry (IHC) in clinical research. However, IHC is not a quantitative assay and is rarely reproducible. We compared VEGF levels in colon cancer by IHC and a quantitative immunoassay on proteins isolated from formalin fixed, paraffin embedded tissues.

**Results:**

VEGF expression was studied by means of a well-based reverse phase protein array (RPPA) and immunohistochemistry in 69 colon cancer cases, and compared with various clinicopathologic factors. Protein lysates derived from formalin fixed, paraffin embedded tissue contained measurable immunoreactive VEGF molecules. The VEGF expression level of well differentiated colon cancer was significantly higher than those with moderately and poorly differentiated carcinomas by immunohistochemistry (*P* = 0.04) and well-based RPPA (*P* = 0.04). VEGF quantification by well-based RPPA also demonstrated an association with nodal metastasis status (*P* = 0.05). In addition, the normalized VEGF value by well-based RPPA correlated (*r* = 0.283, *P* = 0.018). Furthermore, subgroup analysis by histologic type revealed that adenocarcinoma cases showed significant correlation (*r* = 0.315, *P* = 0.031) between well-based RPPA and IHC.

**Conclusions:**

The well-based RPPA method is a high throughput and sensitive approach, is an excellent tool for quantification of marker proteins. Notably, this method may be helpful for more objective evaluation of protein expression in cancer patients.

## Background

The vascular endothelial growth factor (VEGF), one of the key regulators of angiogenesis, is a major target for anti-angiogenic therapy. VEGF overexpression has been associated with vessel invasion [1,2], tumor progression and poor prognosis in several tumor entities, including colorectal cancer [3,4]. It is reported that there is a correlation between VEGF expression and microvessel density [5,6]; therefore, the precise quantification of VEGF expression of clinical specimens is an invaluable factor for understanding clinical outcome, pathophysiological processes or therapeutic responses. In diagnostic pathology setting, tissue samples are mainly preserved as formalin fixed, paraffin embedded (FFPE) tissue blocks for histological examination. Currently, VEGF expressions of clinical FFPE tissue specimens are routinely assessed by immunohistochemistry, however quantification is limited.

Immunochemistry has been used as an adjunct diagnostic method as well as a validation tool of candidate biomarkers for a variety of cancers. Unfortunately, immunohistochemistry is labor intensive and stained specimens must be evaluated by a pathologist one at a time. In addition, several difficult issues remained in evaluation of immunohistochemistry, including the subjective of determination of intensity [7]. Furthermore, immunohistochemical studies with manual scoring are not a quantitative measurement but rather a qualitative assessment. Although image analysis overcomes some of these issues, it lacks normalization to account for differences in tissue handling and processing [8,9]. In order to overcome these limitations of immunohistochemistry, a number of proteomic based technologies have been developed and evaluated in clinical research fields. Although these techniques are generally superior in expression profiling and quantitation of protein changes associated with disease states, each has significant limitations including specimen handling [10]. Many of these methods require frozen tissues or native proteins of starting material required, such limiting their application to FFPE tissue application [11]. Recent advances in techniques for extracting proteins from FFPE tissue sections have been facilitating tissue protein profiling in the clinical proteomics, with varying degrees of success [12,13]. We described a proteomic profiling method which can is applicable to routine clinical FFPE tissue specimens. It is based on combined technologies as a new protein extraction method and a novel protein array platform. We have demonstrated that the array is reliable and a stable proteomic profiling platform for clinical research areas [14].

Here we show that protein expressional assessment by this protein-profiling platform, coupled with protein extraction, correlated with immunohistochemical measurement; therefore, suggesting that it can be used as a substitute method for conventional immunohistochemistry of FFPE tissue specimens. The new proteomic profiling method reported here is, especially in secreted proteins, a sensitive and specific method capable of efficiently unraveling molecular profiles associated with disease status or clinical outcome.

## Results

### Clinicopathologic characteristics of patients

The characteristics of the cases are summarized in Table 1. The ages of the patients ranged from 26 to 89 years (mean, 68.6 years). Thirty-nine patients were men and 30 were women. Six cases were T1 tumors, 9 T2, 43 T3, and 11 T4. Twenty-two patients (32%) had nodal metastasis associated with the primary tumor. Microscopic vascular invasion was identified in 13% of patients (9 cases).

**Table 1:** Characteristics of cases

**Factor**	**Characteristics**	**No. of cases (%)**
**Gender**	Male	39 (56.5%)
	Female	30 (43.5%)
**Differentiation**	Well	3 (4.3%)
	Moderately	31 (44.9%)
	Poorly	35 (50.7%)
**Histologic type**	Adenocarcinoma	46 (66.7%)
	Mucinous carcinoma	23 (33.3%)
**T classification**	T_1_	6 (8.7%)
	T_2_	9 (13.0%)
	T_3_	43 (62.3%)
	T_4_	11 (15.9%)
**N classification**	N_0_	34 (50.0%)
	N_1_	22 (32.4%)
	N_2_	12 (17.6%)
**Vascular invasion**	Absent	60 (87.0%)
	Present	9 (13.0%)

### Immunoreactivity of protein extracted from FFPE tissue specimens

**Figure 1:**
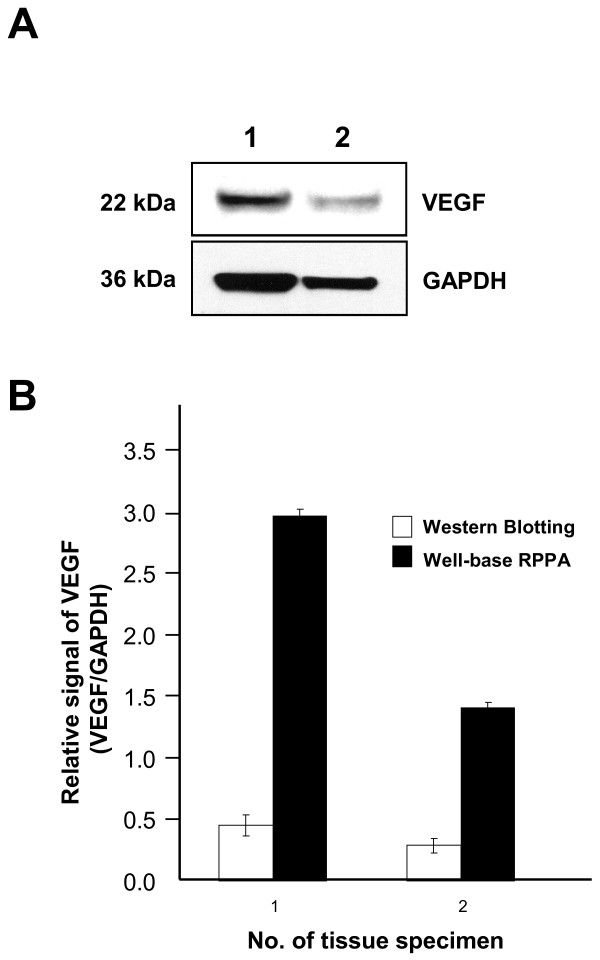
**VEGF expression profiling by well-based reverse phase protein array and western blotting.** We extracted total proteins from 2 different colon FFPE tissue specimens. **(A)** Western blotting shows different VEGF expression pattern. For reliable signal quantitation on same blot, we reprobed the membrane with anti-GAPDH antibodies. Quantative anlysis was performed using the ImageQuant program. **(B)** VEGF and GAPDH expressional signals were measured using well-based reverse phase protein array. Relative expressional signal of VEGF was displayed as a ratio of VEGF to GAPDH. Light bar and black bar represent results of western blotting and well-based reverse phase protein array (RPPA), respectively. The bar graph shows the average ± SD of two independent experiments.

Prior to application of the well-based reverse phase VEGF quantitation, we examined protein quality to determine whether the proteins extracted from archival FFPE human colon specimens were of sufficient quality for well-based reverse-phase protein array (RPPA). We extracted measurable immunoreactive proteins that demonstrated specific signal of the predicted molecular weight by immunoblotting analysis including VEGF and GAPDH, respectively (Figure 1A). Having the immunoreactive proteins, we evaluated whether a well-based RPPA is compatible for VEGF assessment in clinical specimens. Internal control (GAPDH) signals of clinical specimens were strongly correlated with input amount of protein (*R*^*2*^ = 0.9992). Subsequently, we are also confirmed high sensitivity and linearity of using anti-VEGF (*R*^*2*^ = 0.9597), with dynamic range from 0.03 μg to 1.0 μg (Additional file 1: Figure S1). As shown in Figure 1B, relative VEGF signals in two different colon tissues were measured and the ratio of VEGF to GAPDH was calculated. Although there is similar expression pattern between well-based RPPA and immunoblotting, the expression level of novel protein array has a better dynamic range than immunoblotting.

### VEGF immunohistochemical staining

**Figure 2:**
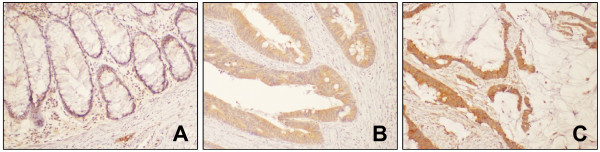
**Immunohistochemical analysis of VEGF expression in colon tissues.** Representative images (final magnification, × 40) of VEGF expression are shown in normal colonic epithelia **(A)**, adenocarcinoma **(B)**, mucinous adenocarcinoma **(C)**. VEGF expression in tumor cells of adenocarcinoma and mucinous carcinoma were strong, while it was weak in normal colonic epithelia.

**Figure 3:**
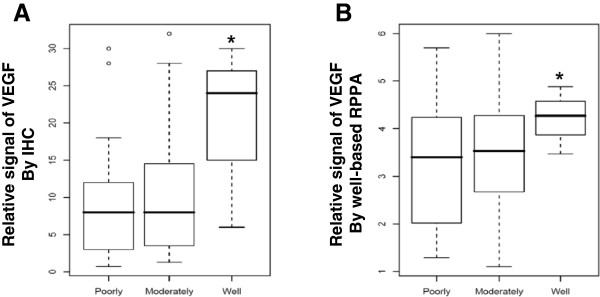
**Relative expression rate of VEGF and its association with histopathologic differentiation.** Box plots of relative expression rates are calculated by immunohistochemistry **(A)** or well-based RPPA **(B)**. Relative expressional signal of VEGF was displayed as a ratio of VEGF to GAPDH. Patients with well differentiated carcinomas had significantly higher expression than those with poorly and moderately differentiated carcinomas. *P < 0.05, ANOVA post-hoc Duncan test.

To determine whether VEGF immunohistochemical expression is associated with clinicopathologic factors in colon cancer, we assessed VEGF expression patterns by semiquantitative analysis of immunohistochemical staining. Representative images of VEGF immunohistochemical staining are illustrated in Figure 2. Results of VEGF immunohistochemistry analysis are summarized in Table 2. Mean histo-score of well differentiated colon cancer (20.0) was significantly higher than those with moderately (10.9) and poorly differentiated (9.1) carcinomas (*P* = 0.04, ANOVA post-hoc Duncan, Figure 3A). However, there were no VEGF expression differences when evaluated with respect to other clinicopathologic factors.

**Table 2:** Results of VEGF expression by immunohistochemical staining and well-based Reverse Phase Protein Array (RPPA)

**Factor**	**Characteristics**	**Quantitation method**			
		**IHC**		**Well-based RPPA**	
		**VEGF**^**a**^	***P*-value**	**VEGF**^**b**^	***P*-value**
**Gender**	Male	8.7	0.07	2.4	0.03^*^
	Female	12.5		3.1	
**Differentiation**	Well	20.0	0.04^*^	4.2	0.04^*^
	Moderately	10.9		2.9	
	Poorly	9.1		2.5	
**Histologic type**	Adenocarcinoma	9.6	0.27	2.8	0.29
	Mucinous carcinoma	11.9		2.5	
**T classification**	T_1_	8.0	0.24	2.1	0.10
	T_2_	6.1		2.9	
	T_3_	11.9		3.0	
	T_4_	9.1		2.3	
**N classification**	N_0_	12.4	0.16	3.1	0.05
	N_1_	8.3		2.5	
	N_2_	8.3		2.3	
**Vascular invasion**	Absent	10.3	0.93	2.8	0.60
	Present	10.0		2.6	

^a^Histo-score was generated as the product of intensity times area.

^b^After normalization with GAPDH level, relative expressional signals were represented as a ratio.

*Significant at the level of *P* < 0.05.

### VEGF quantitation by well-based RPPA

To assess quantitative VEGF scoring, we performed the well-based RPPA with human colon FFPE tissue specimens. Results of VEGF quantitation by the well-based RPPA are summarized in Table 2. Mean relative VEGF expression of well differentiated colon cancer (4.2) was significantly higher than those with moderately (2.9) and poorly differentiated (2.5) carcinomas (*P* = 0.04, ANOVA post-hoc Duncan, Figure 3B). VEGF expression was higher in female patients (3.1) than male patients (2.4, *P* = 0.03). Cases with nodal status N0 (no lymph node metastasis) showed a higher VEGF histo-score (3.1) than what patients with N1 (2.5) or N2 (2.3, *P* = 0.05) showed. There was no VEGF expression difference based on other clinicopathologic factors.

**Figure 4:**
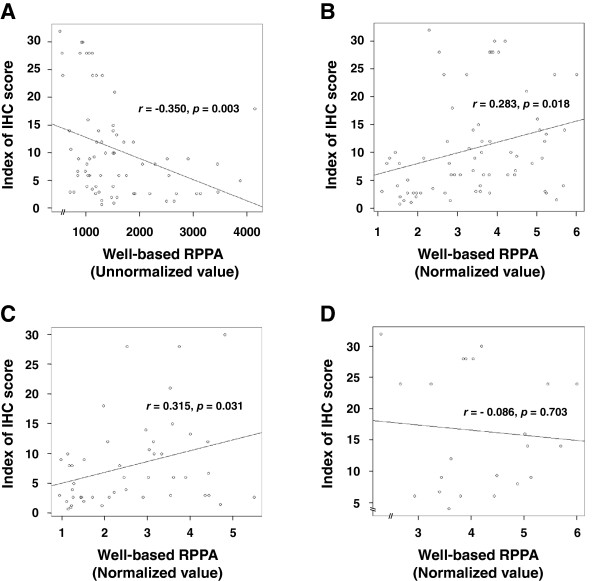
**Reliability of well-based RPPA and its correlation with immunohistochemistry.** Scatter diagrams are presented by unnormalized VEGF value vs. immunohistochemical VEGF score **(A)** and normalized VEGF value vs. IHC VEGF score **(B)**. There was a statistically meaningful correlation between expressional signals in the normalized VEGF value by well-based RPPA and immunohistochemical VEGF score (*r* = 0.283, *P* = 0.018) whereas the unormalized VEGF signal was expressed negative correlation (*r* = -0.350, *P* = 0.003). By histologic subgroup analysis, adenocarcinoma type **(C)** was only showed good correlation (*r* = 0.315, *P* = 0.031) with IHC assessment compare to that of mucinous adenocarcinoma (*r* = -0.086, *P* = 0.703, **D**).

Correlation of VEGF quantitation based on input amount of protein as well as histo-score of VEGF and novel VEGF quantitation assay are summarized on Figure 4. There was an excellent concordance of novel VEGF quantitation assay between 500 ng and 1000 ng of input protein (*P* < 0.0001, *r* = 0.79, Pearson’s correlation, Additional file 1: Figure S2). In addition to the comparison of VEGF quantitation based on input protein, we compared VEGF quantitation based on a new assay with classic “semi-quantitation” of VEGF immunohistochemical expression based on “Histo-score”. Notably, unnormalized VEGF value by well-based RPPA showed negative correlation (*r* = -0.350, *P* = 0.003, Figure 4A) with immunohistochemical assessment, whereas the normalized VEGF value based on GAPDH displayed statistically meaningful correlation (*r* = 0.283, *P* = 0.018, Figure 4B). Furthermore, the adenocarcinoma type showed significantly better correlation (*r* = 0.283, *P* = 0.018, Figure 4C) with immunohistochemical measurement than that of mucinous adenocarcinoma type (*r* = -0.086, *P* = 0.703, Figure 4D) by histological subgroup analysis.

## Discussion

Angiogenesis is one of the hallmarks of cancer and has recently become the target of therapeutic approaches in oncology. Vascular endothelial growth factor (VEGF) was found to be associated with angiogenesis [15-17]. Due to the VEGF expression’s correlation with metastasis and mortality, anti-VEGF therapy shows promise to suppress tumor progression [18]. Based on its importance in regulating tumor angiogenesis, VEGF expression has been assessed in tumor samples via detection of gene amplification or mutation, qualitative RT-PCR, immunohistochemistry, fluorescence *in situ* hybridization, and enzyme-linked immunosorbent assays (ELISA). In clinical research areas, ELISA and immunohistochemistry are commonly employed as VEGF detection assays coupled with serum and FFPE tissue specimens, respectively. VEGF serum levels are generally reported to correlate with the tumor burden and seem to associate with the overall survival of patients with colorectal cancer [19,20]. Werther et al. [21] showed a significant reduced overall survival in patients with high levels of serum VEGF (>533 pg/ml) compared with patients with serum values below this threshold. On the other hand, studies using VEGF immunohistochemistry in FFPE tumor samples are less clear in terms of the prognostic value of VEGF. Ferroni et al. [22] observed a significant association between colorectal tumors with advanced stage and higher VEGF expression by immunohistochemistry. In contrast, Lee et al. [4] and Khorana et al. [23] showed VEGF expression was not associated with survival. There are also conflicting reports between VEGF expression and tumor differentiation. High levels of VEGF expressions were significantly associated with poorly differentiated tumors in colorectal carcinomas [24,25] and soft tissue sarcomas [26]. In this study, however, VEGF expression was elevated in well differentiated tumors in comparison with poorly differentiated ones by immunohistochemistry (*P* = 0.04) and protein array (*P* = 0.04). Similar findings were observed in other studies on colorectal carcinoma [27,28] and esophageal squamous cell carcinoma [29]. Overall the conflicting results of prior studies may be explained by a lack of a standard immunohistochemical method, different standard of interpretation, or differences in studies’ patient populations. Therefore, these problematic environments of VEGF expression assessment have caused some disparity in the reports of VEGF expression as a prognostic marker. Immunohistochemistry, although providing excellent localization, lacks quantification without sophisticated instrumentation and normalization in chromogenic applications.

As a validation study, we employed adenocarcinoma and mucinous carcinoma histologic types for the well-based RPPA assay. Although the adenocarcinoma subgroup showed a good correlation between the well-based RPPA and IHC, the mucinous carcinoma subgroup showed a poor correlation. This difference is due to the cellularity of the samples, where the IHC, un-normalized is an estimate based on a very small number of cells, while the well-based RPPA is a normalized measure. Notably, there is no correlation between tumor cellularity and VEGF expressional signals by well-based RPPA (Spearman’s rho = 0.088, *P* = 0.471) whereas the IHC values shown a statistical correlation with the cellularity (Spearman’s rho = 0.700, *P* < 0.0001). To date, all immunohistochemical studies of VEGF quantitation in colon cancer tissues are limited to tumor areas and do not reflect the contribution of VEGF in adjacent tissue. Since VEGF is a secreted protein, its presence not only in tumor cells but also in surrounding matrix and cellular membranes has hampered its analysis by immunohistochemistry [30,31]. Therefore, lysates of whole sections would better represent the complex VEGF expression pattern.

Formalin fixation results in protein cross-linking. Moreover, the protein quality of FFPE tissue is impacted by additional pre-analytical variable factors including warm ischemia, fixation time and tissue processing conditions [32]. Although it is typical for extracted proteins from FFPE tissue to show a "smear" on SDS-PAGE, the extent of the smear is related to the specimen processing conditions, with greater "smearing" indicates different degradation levels for each tissue sample. In this study, we used GAPDH protein signal as an internal control to measure the protein quality from each tissue sample. The GAPDH protein has a relatively small molecular weight and is relatively well-reserved in FFPE tissue [14]. In this context, the VEGF signal was double normalized with total amount of protein and the GAPDH signal. By employing the normalization tool by GAPDH, our methodology has an advantage that can evaluate VEGF value without the risk of low reliability and poor validity in the current immunohistochemical VEGF assessment.

## Conclusion

In conclusion, we revealed a better correlation and less scattering using the novel protein array methodology in tissue lysates of FFPE tissue. In addition, we showed the new approach could be used for protein profiling analysis in FFPE tissue, with quantification and normalization tools. As a research tool, this method offers an additional great tool for assessment of VEGF expression in translational medicine.

## Methods

### Patients and tumor samples

The study subjects were composed of 69 patients with surgically resected colon cancer at RWTH Aachen University, Aachen, Germany. Medical records were reviewed to obtain data including age, gender of patients, cancer stage, tumor differentiation, cell type, lymphovascular invasion and lymph node metastasis. Tissue samples were collected from patients who had signed informed consent forms, which was approved by the institutional review board of the RWTH Aachen University Hospital. This study was additionally approved by the Office of Human Subjects Research at the NIH. Information on post-operative radiation and/or chemotherapy, and outcome of patients were not available.

### Protein extractions from FFPE tissue sections

Protein extraction from two 10 μm FFPE tissue sections was performed as previously described [14]. This methodology has the advantage of being compatible with archival tissue and does not require deparaffinization. Briefly, sections were trimmed of excess wax and homogenized using a Disposable Pellet Mixer in 200 μl protein extraction solution [1x high pH Antigen retrieval buffer (pH 9.9) (Dako, Carpinteria, CA), 1% NaN_3_, 1% SDS, 10% glycerol and protease inhibitor (1 tablet/25 ml, Roche)], followed by incubation for 15 min at 115°C within a pressure cooker (Dako). After incubation, the tissue lysates were centrifuged at 13,000 rpm for 30 min at 4°C. The supernatants were collected and stored at -20°C. Total protein concentrations were measured with the BCA Protein Assay kit (Pierce Biotechnology, Rockford, IL).

### SDS-PAGE and western blotting

The protein extracted from FFPE tissue sections was resolved by 4-12% NuPAGE® Novex Bis-Tris polyacrylamide gel, and electroblotted to nitrocellulose membrane using iBlot™ Dry Blotting System (Invitrogen, Carlsbad, CA). The membranes were blocked with 5% nonfat dry milk in TBST (50 mM Tris, pH 7.5, 150 mM NaCl, 0.05% Tween-20) for 1 h, washed, and subsequently incubated overnight at 4°C in TBST with 5% BSA containing the following antibodies; anti-VEGF (NeoMarkers, Fremont, CA, 1:50) and anti-GAPDH (Calbiochem, Gibbstown, NJ, 1:5000). Specific molecules were detected with horseradish peroxidase-labeled anti-mouse antibodies (Chemicon International, Temecula, CA) and enhanced with SuperSignal Chemiluminescence kit (Pierce Biotechnology). Signals were detected on KODAK BIOMAX MR X-ray film (Kodak, Rochester, NY). Quantative analysis of the western blotting was performed using ImageQuant (*Ver.* 5.2, Molecular Dynamics, Sunnyvale, CA).

### VEGF quantitation by well-based reverse phase protein array

VEGF expression signals from archival FFPE tissues were measured as previously reported [14]. Briefly, five microliters of protein extract from FFPE tissue specimen, at predetermined protein concentrations, were added to Meso Scale Discovery (MSD, Gaithersburg, MD) Multi-Spot™ plates (MA2400 96 HB Plate). The plate was allowed to dry at room temperature for 90 min, and the plates were subsequently further incubated for 30 min at 37°C. The antigen-coated plates were preincubated with 5% BSA in PBST for 60 min at RT before primary antibody reactions. Anti-VEGF (Neomarkers) and anti-GAPDH (Calbiochem) were diluted 1:200 and 1:1000 with 5% BSA in PBST, and then incubated overnight at 4°C. After washing with PBST, the plates were incubated for 1 h with goat anti-mouse SULFO-TAG™ antibodies at a dilution of 1:1000 (0.5 μg/ml) with 5% nonfat milk in PBST. The plates were then aspirated and washed three times with PBST. Finally, MSD-T read buffer was added to the plates and they were read on the MSD Sector Imager 2400 reader (Meso Scale Discovery). BSA coated wells were included on each plate as a control for non-specific binding effects. The values from non-specific wells were subtracted from all standards samples to calculate actual value. Two independent experiments were performed with triplicates.

### Immunohistochemistry and scoring

Tissue sections were deparaffinized and rehydrated in xylene and serial alcohol solutions, respectively. Endogenous peroxidase was blocked by incubation in 3% H_2_O_2_ for 10 min. The antigen retrieval was performed in a steam pressure cooker with prewarmed antigen retrieval buffer pH 9 (Dako) at 95°C, for 20 min. To minimize non-specific staining, sections were incubated with protein block (Dako) for 15 min. Anti-VEGF antibody (Neomarkers, 1:50) were incubated for 60 min at RT. After secondary incubation of Envision + kit/horseradish peroxidase (Dako), substrate chromogen was added and specimens were lightly counterstained with hematoxylin.

Protein expression for tumour cells was measured over the full tissue specimen using the weighted histo-score method [33]. The weighted histo-score grades staining intensity as 0 (negative), 1+ (weak), 2+ (moderate), or 3+ (strong), then multiplication of percentage of tumor cells within each category. Furthermore, three additional different areas were scored by percentage and intensity: normal colon epithelium, adjacent tissue and surrounding extracellular matrix. Two independent pathologists (TB, SMH) scored slides blindly and separately. For those cases with discrepancy amongst pathologists, a decision was made based on the consensus opinion.

### Statistical analysis

Statistical analyses were performed using SPSS (version 19, Chicago, IL) and R (http://www.r-project.org). Comparison of means was performed using Student’s *t*-test, Mann-Whitney, or one-way ANOVA tests. Linear regression analysis was performed to examine the relationship of VEGF expression by immunohistochemical staining and VEGF quantitation by well-based RPPA. A *p*-value less than 0.05 was considered statistically significant.

## Abbreviations

ELISA: Enzyme-linked immunosorbent assays; FFPE: Formalin-fixed and paraffin-embedded; GAPDH: Glyceraldehyde-3-phospate dehydrogenase; IHC: Immunohistochemistry; RPPA: Reverse phase protein array; VEGF: Vascular endothelial growth factor.

## Competing interests

The authors declare that they have no competing interests.

## Authors’ contributions

J-YC and SMH conceived the study and devised the experimental design for novel protein assays. J-YC, TB, and DK performed experiments. S-MH, S-HE, HC, TB, J-YC and SMH performed data analysis for experiments and clinical records. J-YC, TB and S-MH drafted the final version of the manuscript and figure legends. SMH revised the figures, added critical content to the discussion and was responsible in revising all portions of the submitted portion of the manuscript. All authors read and approved the final manuscript.

## Supplementary Material

Additional file 1: Figure S1 Protein expression profiling by well-based RPPA. **Figure S2.** Reliability of well-based RPPA for VEGF assessment in FFPE tissue lysates. Click here for file

## References

[B1] Zebrowski BK Liu W Ramirez K Akagi Y Mills GB Ellis LM Markedly elevated levels of vascular endothelial growth factor in malignant ascites Ann Surg Oncol 1999 6 373 378 10.1007/s10434-999-0373-0 10379858

[B2] Zebrowski BK Yano S Liu W Shaheen RM Hicklin DJ Putnam JB Jr Ellis LM Vascular endothelial growth factor levels and induction of permeability in malignant pleural effusions Clin Cancer Res 1999 5 3364 3368 10589746

[B3] Takahashi Y Kitadai Y Bucana CD Cleary KR Ellis LM Expression of vascular endothelial growth factor and its receptor, KDR, correlates with vascularity, metastasis, and proliferation of human colon cancer Cancer Res 1995 55 3964 3968 7664263

[B4] Lee JC Chow NH Wang ST Huang SM Prognostic value of vascular endothelial growth factor expression in colorectal cancer patients Eur J Cancer 2000 36 748 753 10.1016/S0959-8049(00)00003-4 10762747

[B5] Perrone G Vincenzi B Santini D Verzi A Tonini G Vetrani A Rabitti C Correlation of p53 and bcl-2 expression with vascular endothelial growth factor (VEGF), microvessel density (MVD) and clinico-pathological features in colon cancer Cancer Lett 2004 208 227 234 10.1016/j.canlet.2003.11.032 15142682

[B6] Cristi E Perrone G Toscano G Verzi A Nori S Santini D Tonini G Vetrani A Fabiano A Rabitti C Tumour proliferation, angiogenesis, and ploidy status in human colon cancer J Clin Pathol 2005 58 1170 1174 10.1136/jcp.2004.025536 16254106 PMC1770760

[B7] Taylor CR The total test approach to standardization of immunohistochemistry Arch Pathol Lab Med 2000 124 945 951 10888767 10.5858/2000-124-0945-TTTATS

[B8] Taylor CR Levenson RM Quantification of immunohistochemistry–issues concerning methods, utility and semiquantitative assessment II Histopathology 2006 49 411 424 10.1111/j.1365-2559.2006.02513.x 16978205

[B9] Takikita M Chung JY Hewitt SM Tissue microarrays enabling high-throughput molecular pathology Curr Opin Biotechnol 2007 18 318 325 10.1016/j.copbio.2007.05.007 17643281

[B10] Miyaji T Hewitt SM Liotta LA Star RA Frozen protein arrays: a new method for arraying and detecting recombinant and native tissue proteins Proteomics 2002 2 1489 1493 10.1002/1615-9861(200211)2:11<1489::AID-PROT1489>3.0.CO;2-8 12442248

[B11] Chung JY Braunschweig T Baibakov G Galperin M Ramesh A Skacel M Gannot G Knezevic V Hewitt SM Transfer and multiplex immunoblotting of a paraffin embedded tissue Proteomics 2006 6 767 774 10.1002/pmic.200401343 16400680

[B12] Shi SR Liu C Balgley BM Lee C Taylor CR Protein extraction from formalin-fixed, paraffin-embedded tissue sections: quality evaluation by mass spectrometry J Histochem Cytochem 2006 54 739 743 10.1369/jhc.5B6851.2006 16399996

[B13] Becker KF Schott C Hipp S Metzger V Porschewski P Beck R Nahrig J Becker I Hofler H Quantitative protein analysis from formalin-fixed tissues: implications for translational clinical research and nanoscale molecular diagnosis J Pathol 2007 211 370 378 10.1002/path.2107 17133373

[B14] Chung JY Lee SJ Ylaya K Braunschweig T Traicoff JL Hewitt SM A well-based reverse-phase protein array applicable to extract from formalin-fixed paraffin-embedded tissue Proteomics Clin Appl 2008 2 1539 1547 10.1002/prca.200800005 21136801 PMC3777740

[B15] Kawasaki H Toyoda M Shinohara H Okuda J Watanabe I Yamamoto T Tanaka K Tenjo T Tanigawa N Expression of survivin correlates with apoptosis, proliferation, and angiogenesis during human colorectal tumorigenesis Cancer 2001 91 2026 2032 10.1002/1097-0142(20010601)91:11<2026::AID-CNCR1228>3.0.CO;2-E 11391581

[B16] Toi M Matsumoto T Bando H Vascular endothelial growth factor: its prognostic, predictive, and therapeutic implications Lancet Oncol 2001 2 667 673 10.1016/S1470-2045(01)00556-3 11902537

[B17] Prall F Gringmuth U Nizze H Barten M Microvessel densities and microvascular architecture in colorectal carcinomas and their liver metastases: significant correlation of high microvessel densities with better survival Histopathology 2003 42 482 491 10.1046/j.1365-2559.2003.01610.x 12713626

[B18] Cascinu S Staccioli MP Gasparini G Giordani P Catalano V Ghiselli R Rossi C Baldelli AM Graziano F Saba V Muretto P Catalano G Expression of vascular endothelial growth factor can predict event-free survival in stage II colon cancer Clin Cancer Res 2000 6 2803 2807 10914727

[B19] Akbulut H Altuntas F Akbulut KG Ozturk G Cindoruk M Unal E Icli F Prognostic role of serum vascular endothelial growth factor, basic fibroblast growth factor and nitric oxide in patients with colorectal carcinoma Cytokine 2002 20 184 190 10.1006/cyto.2002.1993 12543084

[B20] Gunsilius E Tschmelitsch J Eberwein M Schwelberger H Spizzo G Kahler CM Stockhammer G Lang A Petzer AL Gastl G In vivo release of vascular endothelial growth factor from colorectal carcinomas Oncology 2002 62 313 317 10.1159/000065062 12138238

[B21] Werther K Christensen IJ Nielsen HJ A consistent shift in VEGF determinations between two different ELISA batch numbers Br J Cancer 2003 89 420 10.1038/sj.bjc.6601123 12865938 PMC2394259

[B22] Ferroni P Palmirotta R Spila A Martini F Formica V Portarena I Del Monte G Buonomo O Roselli M Guadagni F Prognostic value of carcinoembryonic antigen and vascular endothelial growth factor tumor tissue content in colorectal cancer Oncology 2006 71 176 184 10.1159/000106072 17652942

[B23] Khorana AA Ryan CK Cox C Eberly S Sahasrabudhe DM Vascular endothelial growth factor, CD68, and epidermal growth factor receptor expression and survival in patients with Stage II and Stage III colon carcinoma: a role for the host response in prognosis Cancer 2003 97 960 968 10.1002/cncr.11152 12569594

[B24] Zheng S Han MY Xiao ZX Peng JP Dong Q Clinical significance of vascular endothelial growth factor expression and neovascularization in colorectal carcinoma World J Gastroenterol 2003 9 1227 1230 12800229 10.3748/wjg.v9.i6.1227 PMC4611789

[B25] Hanrahan V Currie MJ Gunningham SP Morrin HR Scott PA Robinson BA Fox SB The angiogenic switch for vascular endothelial growth factor (VEGF)-A, VEGF-B, VEGF-C, and VEGF-D in the adenoma-carcinoma sequence during colorectal cancer progression J Pathol 2003 200 183 194 10.1002/path.1339 12754739

[B26] Chao C Al-Saleem T Brooks JJ Rogatko A Kraybill WG Eisenberg B Vascular endothelial growth factor and soft tissue sarcomas: tumor expression correlates with grade Ann Surg Oncol 2001 8 260 267 10.1007/s10434-001-0260-9 11314944

[B27] Uner A Ebinc FA Akyurek N Unsal D Mentes BB Dursun A Vascular endothelial growth factor, c-erbB-2 and c-erbB-3 expression in colorectal adenoma and adenocarcinoma Exp Oncol 2005 27 225 228 16244586

[B28] Abdou AG Aiad H Asaad N Abd El-Wahed M Serag El-Dien M Immunohistochemical evaluation of vascular endothelial growth factor (VEGF) in colorectal carcinoma J Egypt Natl Canc Inst 2006 18 311 322 18301455

[B29] Inoue K Ozeki Y Suganuma T Sugiura Y Tanaka S Vascular endothelial growth factor expression in primary esophageal squamous cell carcinoma. Association with angiogenesis and tumor progression Cancer 1997 79 206 213 10.1002/(SICI)1097-0142(19970115)79:2<206::AID-CNCR2>3.0.CO;2-I 9010092

[B30] Tischer E Mitchell R Hartman T Silva M Gospodarowicz D Fiddes JC Abraham JA The human gene for vascular endothelial growth factor. Multiple protein forms are encoded through alternative exon splicing J Biol Chem 1991 266 11947 11954 1711045

[B31] Fukumura D Xavier R Sugiura T Chen Y Park EC Lu N Selig M Nielsen G Taksir T Jain RK Seed B Tumor induction of VEGF promoter activity in stromal cells Cell 1998 94 715 725 10.1016/S0092-8674(00)81731-6 9753319

[B32] Hewitt SM Lewis FA Cao Y Conrad RC Cronin M Danenberg KD Goralski TJ Langmore JP Raja RG Williams PM Palma JF Warrington JA Tissue handling and specimen preparation in surgical pathology: issues concerning the recovery of nucleic acids from formalin-fixed, paraffin-embedded tissue Arch Pathol Lab Med 2008 132 1929 1935 19061293 10.5858/132.12.1929

[B33] Kirkegaard T Edwards J Tovey S McGlynn LM Krishna SN Mukherjee R Tam L Munro AF Dunne B Bartlett JM Observer variation in immunohistochemical analysis of protein expression, time for a change? Histopathology 2006 48 787 794 10.1111/j.1365-2559.2006.02412.x 16722926

