# Supplementary material for: Assessment of vascular endothelial growth factor in formalin fixed, paraffin embedded colon cancer specimens by means of a well-based reverse phase protein array
Source: Proteome Sci. 2014 May 13;12:27. doi: 10.1186/1477-5956-12-27 (PMC4039052; doi:10.1186/1477-5956-12-27)
Supplement: Additional file 1: Figure S1 — Protein expression profiling by well-based RPPA. Figure S2. Reliability of well-based RPPA for VEGF assessment in FFPE tissue lysates. [file 1477-5956-12-27-S1.doc]

**Supplementary figures**


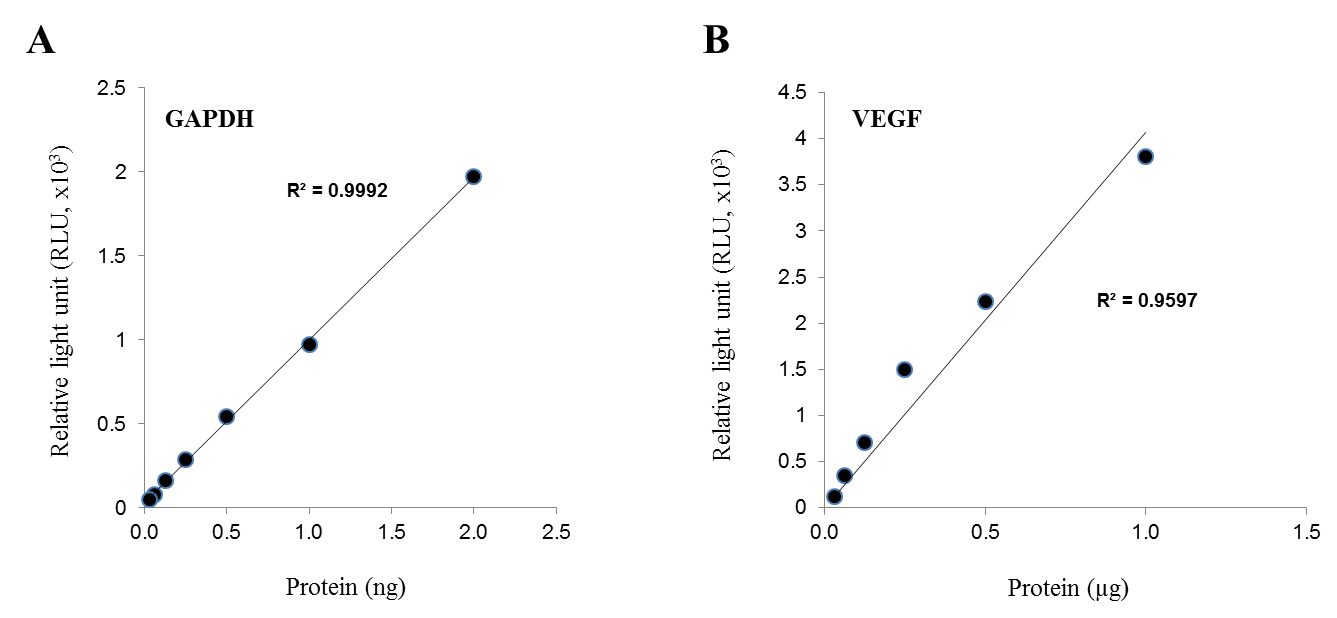


**Supplementary Figure 1 Protein expression profiling by well-based RPPA.** Standard curve of GAPDH (A, *R*^2^=0.999) and VEGF (A, *R*^2^=0.960) by well-based RPPA.


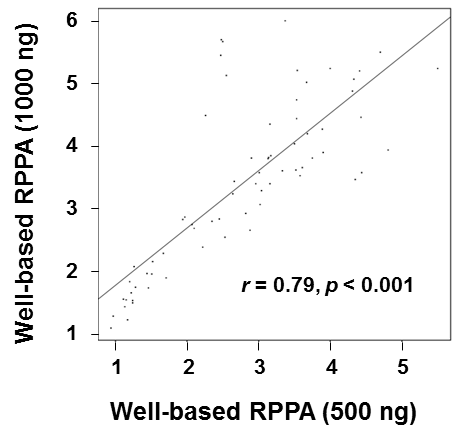


**Supplementary Figure 2 Reliability of well-based RPPA for VEGF assessment in FFPE tissue lysates.** Well-based RPPA had showed a great correlation between 500 ng and 1000 ng (*r* = 0.79, *P*<0.001. Pearson’s correlation).
